# Cyanobacteria as a Promising Alternative for Sustainable Environment: Synthesis of Biofuel and Biodegradable Plastics

**DOI:** 10.3389/fmicb.2022.939347

**Published:** 2022-07-13

**Authors:** Preeti Agarwal, Renu Soni, Pritam Kaur, Akanksha Madan, Reema Mishra, Jayati Pandey, Shreya Singh, Garvita Singh

**Affiliations:** Department of Botany, Gargi College, University of Delhi, New Delhi, India

**Keywords:** biodegradable, bioplastics, biofuel, biopolymer, clean energy, polyhydroxyalkanoates, sustainable

## Abstract

With the aim to alleviate the increasing plastic burden and carbon footprint on Earth, the role of certain microbes that are capable of capturing and sequestering excess carbon dioxide (CO_2_) generated by various anthropogenic means was studied. Cyanobacteria, which are photosynthetic prokaryotes, are promising alternative for carbon sequestration as well as biofuel and bioplastic production because of their minimal growth requirements, higher efficiency of photosynthesis and growth rates, presence of considerable amounts of lipids in thylakoid membranes, and cosmopolitan nature. These microbes could prove beneficial to future generations in achieving sustainable environmental goals. Their role in the production of polyhydroxyalkanoates (PHAs) as a source of intracellular energy and carbon sink is being utilized for bioplastic production. PHAs have emerged as well-suited alternatives for conventional plastics and are a parallel competitor to petrochemical-based plastics. Although a lot of studies have been conducted where plants and crops are used as sources of energy and bioplastics, cyanobacteria have been reported to have a more efficient photosynthetic process strongly responsible for increased production with limited land input along with an acceptable cost. The biodiesel production from cyanobacteria is an unconventional choice for a sustainable future as it curtails toxic sulfur release and checks the addition of aromatic hydrocarbons having efficient oxygen content, with promising combustion potential, thus making them a better choice. Here, we aim at reporting the application of cyanobacteria for biofuel production and their competent biotechnological potential, along with achievements and constraints in its pathway toward commercial benefits. This review article also highlights the role of various cyanobacterial species that are a source of green and clean energy along with their high potential in the production of biodegradable plastics.

## Introduction

There is an unprecedented increase in carbon footprint due to increased toxicant levels in the atmosphere and excessive use of fossil fuels as well as their plastic derivatives, which are being discarded in landfill sites and will persist in the environment for more than the next 100 years. It has drastically posed new environmental challenges for nature and the survival of living organisms. Rapid climate change, along with ever-increasing billions of tons of plastic products in almost every inch of the planet, has posed serious threats to the Earth and would contribute to a massive global environmental hazard (Borrelle et al., 2020; Silva et al., 2020). The marine ecosystem is facing severe impacts because of accumulation of plastic debris with minimal degradation properties (Jambeck et al., 2015). Continuous accumulation of plastics in various strata of water bodies has led to the origin of a new ecosystem acknowledged as the “plastisphere.” This is an outcome of the long shelf life and hydrophobic nature of non-degradable plastics being accumulated as debris, which could be the cause of replacement of natural ecosystems leading to unknown natural disasters (Zettler et al., 2013; Bergmann et al., 2017). Another outcome is the fragmentation of plastics to microplastics to the level that they mark a presence in the food chain imposing a threat to the whole ecosystem (Chen, 2015; Giacovelli and Environment: Technology for Environment, 2018). This is further being aggravated because of the prevailing COVID-19 situation across the globe, which has led to an uncalculated increase in single-use plastics in various forms and is another upcoming threat we are going to observe in the environment in the coming times (Ammendolia et al., 2021; Mejjad et al., 2021). Moreover, the energy crisis that the world is facing because of continuous depletion of fossil fuels could spark a misbalance in the world economy and progress. This further leads to new challenges to attain environmental goals wherein an unfiltered usage of non-biodegradable plastics from various unconventional resources is another major issue for a sustainable future. A recent study from IPCC (2014), the Inter-governmental Panel on Climate Change, projects a worrisome image wherein climate change could result in more severe impacts if we fail to abate GHG (greenhouse gas) emission in the near future. Biodiversity is shrinking as a result of prominent desert spread and choking of warmer oceans with plastic waste. In reality, the plastic industry consumes around 6% of world oil and is anticipated to grow up to 20% by 2050, as stated by IEA (International Energy Agency, 2017), which needs attention (Giacovelli and Environment: Technology for Environment, 2018). A continuous assessment conducted by World Economic Forum has put up an image wherein plastic production across the globe will further increase by 2-fold in the coming 20 years. This production of plastic would be accompanied by generation of enormous amounts of GHG emission. Combustion of fossil fuels is estimated to have caused a rise in atmospheric CO_2_ from 300 to 400 ppm in the past century (Jambeck et al., 2015). These elevated carbon dioxide levels have led to a huge loss of glacial mass in polar regions such as Greenland and Antarctic belts, portraying unrequited human behavior toward the environment. Apart from these alarming scenarios, climate change is likely to be accompanied by rising sea levels that could cause higher precipitation and submergence in various regions (Pachauri et al., 2014).

There is an acute need to identify new sustainable substrates that could pace up with both energy demands in the form of biofuels as commercial products and biodegradable plastics as clean and green resources. Thoughtful calculations to attain carbon neutrality have predicted that cutting down on fossil fuel usage by 6–7 percent every year until 2030 is the only way to stop cataclysmic after-effects generated by fossil fuels and non-degradable and non-recyclable plastics. The environmental and economic costs associated with fossil fuel consumption both for energy requirements and commercial resources such as plastics have been garnering immense criticism, pushing a massive interest in biofuels and bioplastics for a sustainable future.

With great success, biofuels and bioplastics have been produced commercially from terrestrial plants (both monocots and dicots). However, the cultivation of these crops for biofuel or bioplastic derivation creates an immense competition for agricultural land against edible crops. In contrast to terrestrial plants currently being exploited for biofuel and bioplastic production, bacteria, yeast, and cyanobacteria prove to be more efficient and feasible alternatives. Bacterial cultivation, being a heterotrophic model, entails higher expenses in lieu of the costly input of organic nutrients required to accumulate crucial metabolites. The rate-limiting usage of organic substrates for the fermentation process in the production of bacterial bioplastic is one of the major cost additions, the second-largest expense is the energy-intensive procedures involved in polyhydroxyalkanoate (PHA) extraction. The bacterial, heterotrophic model for bioplastic production is therefore far from becoming economically feasible (Markou and Nerantzis, 2013; Karan et al., 2019).

**Figure 1:**
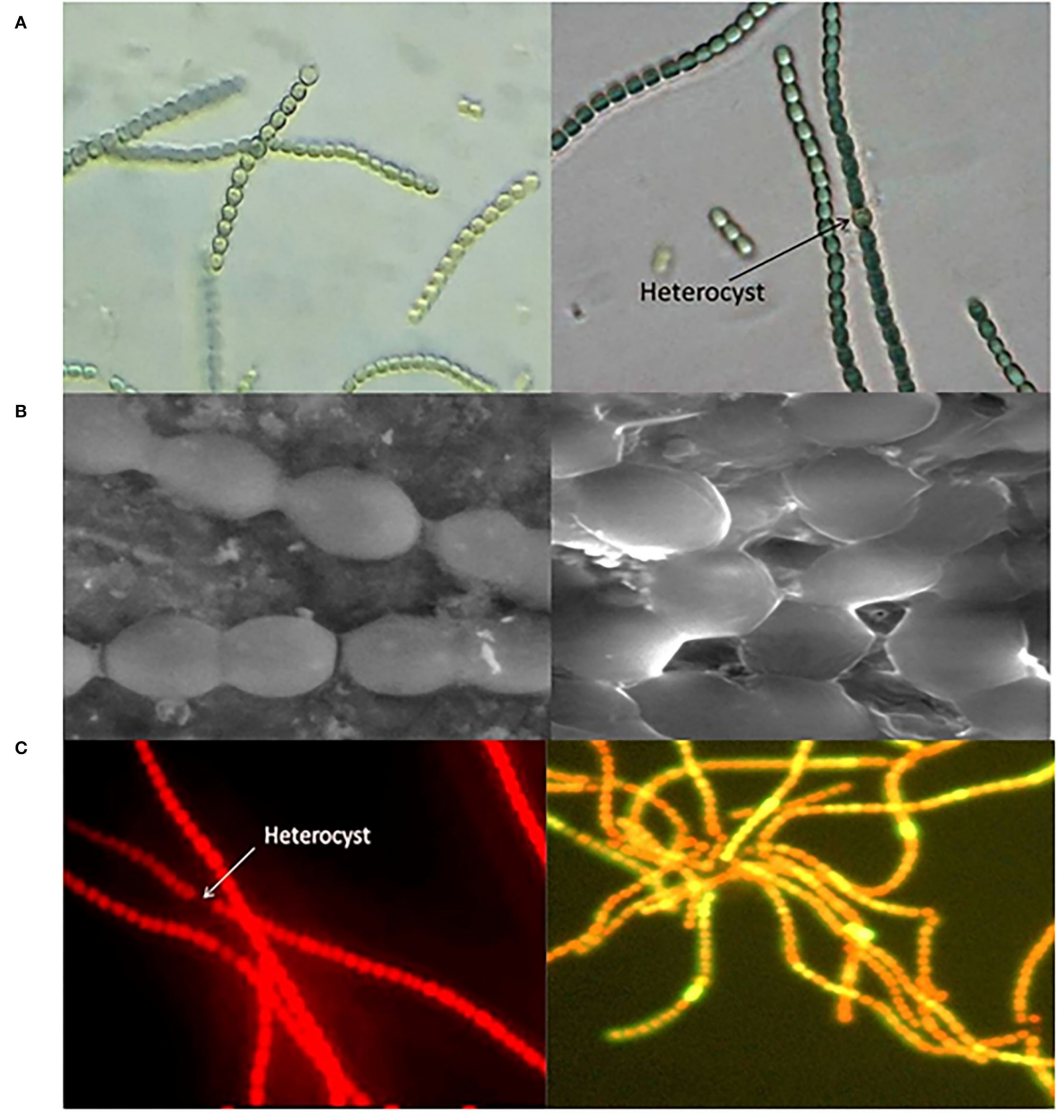
Mic of Cyanobacterium *Anabaena* sp. **(A)** Phase contrast microscopic images, **(B)** scanning electron microscopic (SEM) images, and **(C)** fluorescence microscopic image showing auto-fluorescent pigments.

However, now, the world is not only talking about reducing CO_2_ but also about employing microalgae and cyanobacteria for the biological fixation of atmospheric CO_2_, which provides an efficient and feasible strategy to sequester excess carbon. Cyanobacteria are being studied for biological life support systems in various forms and means. Growth patterns, cell assembly, and targeted genetic modulations are being conducted in order to get the desired industrial products (Rosenboom et al., 2022). Several common cyanobacterial species such as *Anabaena cylindrica, Anabaena muscorum, Anabaena doliolum*, and *Synechocystis* sp. have been tested for their potential role in the production of various industrial compounds including biofuel and bioplastics. Various identification measures including phase contrast microscopy and scanning electron microscopy (SEM) along with fluorescence microscopic images (Figure 1) reveal their nature and alignment of cells and elaborate autoflourescent compounds as phycobiliproteins and chlorophylls. These organisms are quite promising, with biofixation efficiency estimated to be ~10-fold more than that of terrestrial plants (Cheah et al., 2015; Cuellar-Bermudez et al., 2015). In the last 15 years, from the very first pivotal steps of detecting chemicals of industrial assets expressed from exogenous genes in cyanobacteria, algal biotechnology has advanced in several prospects. Through robust studies on photosynthetic pathways in cyanobacteria, desired products on a commercial scale for understanding the metabolic machinery of host organisms have been attained (Oliver et al., 2013). Next, there is a need to overcome various challenges posed for sustainable biofuel and bioplastic production by cyanobacteria. Engineering cyanobacterial strains for enhancing yield is challenging as the metabolic pathway involves oxygen-sensitive enzymes. Selection and optimization of strains that possess the ability to metabolize both fatty acid content and precursors, play a significant role in commercializing cyanobacterium-derived biofuels and bioplastics for a sustainable future. As mentioned earlier, these organisms show a much higher photosynthetic efficiency of up to 10% than terrestrial plants possessing an efficiency of 4% (Lewis and Nocera, 2006); furthermore, they require minimal land and nutrient input. To attain a sustainable economy, there is an urgent need for the replacement of fossil fuel substrates for various commercial products such as plastics and other polymers. An interdisciplinary approach involving an understanding of synergistic actions and selection of feedstock biomass to useful compounds like biofuels or bioplastics is ultimately the only roadmap that could help in attaining a better environmental prospect.

This review aims to highlight the potential role of cyanobacteria as a green sustainable methodology for biofuel and bioplastic production and enhance their yield by modifying metabolic pathways by genetic engineering. This review also features scope and constraints related to large-scale industrial production of biofuel and bioplastics. The dynamic metabolic versatility of cyanobacteria offers potential to overcome hurdles related to first-generation biofuel that utilizes plant resources as a substrate which has large land and water requirements. This has put considerable trust and interest in exploring cyanobacteria, a step toward sustainability for biofuels and bioplastics. The purpose of this study, therefore, is to portray a suitable approach toward the production of green energy as biofuels (generations III and IV) and bioplastics for a sustainable environment utilizing cyanobacteria as a biomass source.

## Cyanobacteria: A Sustainable Biofactory for Biofuel and Bioplastic Production

**Figure 2:**
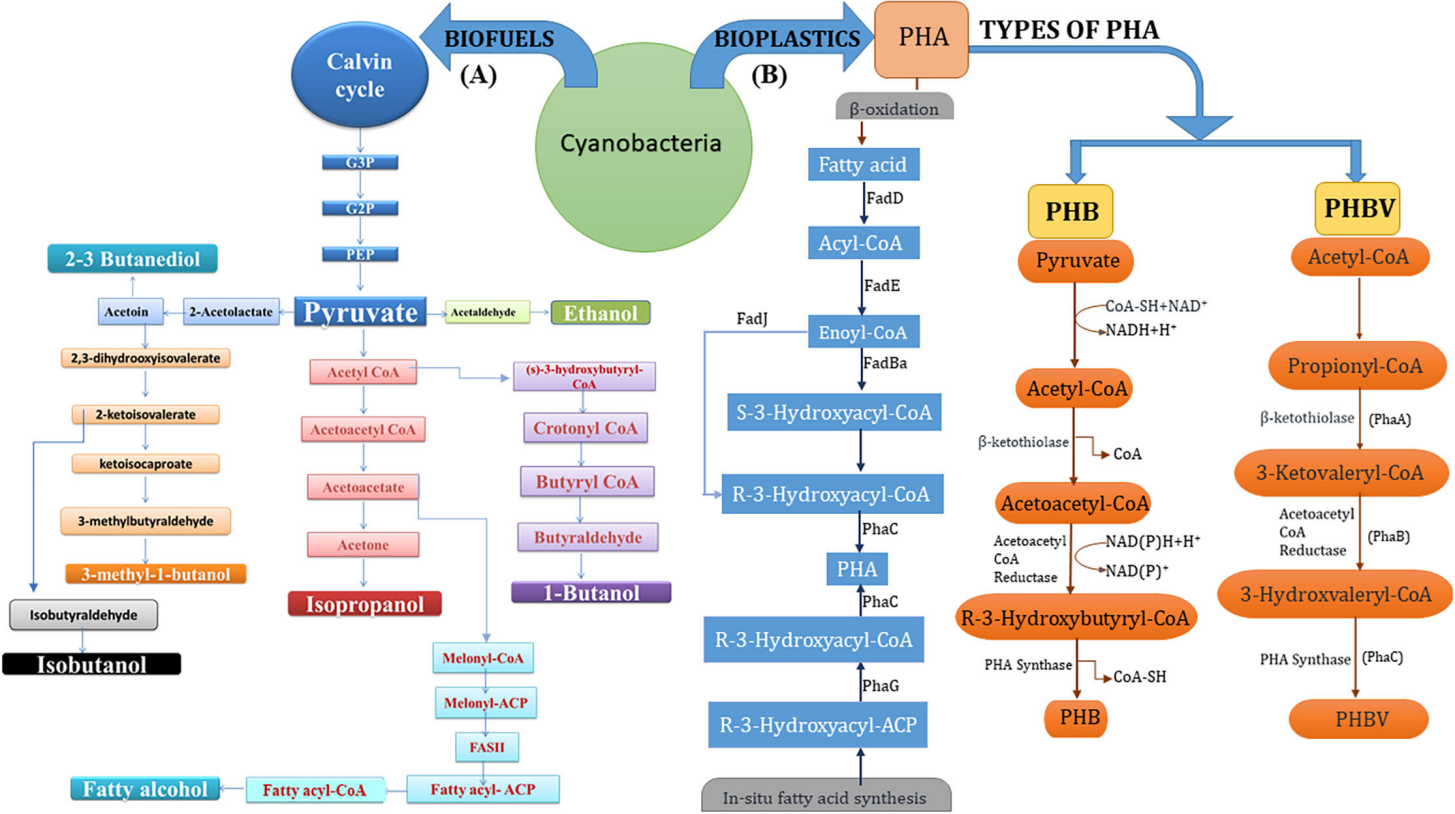
**(A)** Schematic representation of cyanobacterial metabolic pathways leading to biofuel (bioethanol) derivatives *viz*. ethanol, butanol, isobutanol, isopropanol, 2,3-butanediol, and fatty alcohol; all derived from Calvin cycle and associated intermediate biomolecules (especially pyruvate). **(B)** Synthesis of PHA copolymers (PHB and PHBV) through enzyme-mediated 3-step pathway i.e., condensation, reduction, and polymerization with precursor acetyl-CoA molecules and through fatty acid β-oxidation pathway.

Cyanobacteria, prokaryotic photosynthetic organisms and ancestors of present-day chloroplasts, are extremely potential microbes that are being selected for their efficiency for production of biofuels and biodegradable plastics because of their promising attributes such as mitigation strategy for atmospheric CO_2_ absorption and fixation, utilizing it for its growth in adverse climatic conditions such as saline water and barren unfertile land, with a potential to fix atmospheric nitrogen. Their high specific growth rate, abundant fatty acid and oil content, and other active metabolites make them a better-suited alternative to other resources (Gouveia and Oliveira, 2009). Figure 2 illustrates a schematic representation of the innate efficiency of cyanobacteria to convert nutrients and CO_2_ into high-value cell constituents that can be harnessed for commercial-scale production of industrially and commercially relevant chemicals like biofuels (especially bioethanol and butanol) and materials like bioplastics (PHB and PHBV).

Thus, among various resources available for biofuel and bioplastic production, cyanobacteria have long been considered as energy-rich sources because of production of diacylglycerol (DAG) and triacylglycerol (TAG) that can be utilized as precursors of biodiesel. The availability of genetic sequence information of these microbes further makes them efficient in being utilized for generation of renewable biofuels (Wijffels et al., 2013; Halfmann et al., 2014; Savakis and Hellingwerf, 2015). According to research conducted in the past, cyanobacteria and algae produce a wide range of chemical intermediates and hydrocarbons that act as precursors for biofuels. As a result, cyanobacterium-derived fuel holds a great promise as a possible replacement for present fossil-fuel-derived goods (Nozzi et al., 2013). The biomass obtained from cyanobacteria can be utilized as a food source or as a variety of feedstock, and antioxidants, coloring agents, medicines, and bioactive chemicals are just a few of the significant biomolecules that may be extracted from cyanobacteria. Various types of biofuels obtained from cyanobacteria include bioethanol, isobutanol, ethylene, 1-butanol, biomethane, biodiesel, and biohydrogen (Schenk et al., 2008).

Cyanobacteria also possess the metabolism for producing economically useful and sustainable biopolymer polyhydroxyalkanoates, PHAs, and their various copolymers including polyhydroxybutyrate (PHB). The biopolymeric PHB exhibits material properties similar to polypropylene, a conventional plastic derived from fossil fuels such as petroleum. However, in contrast to conventional plastics, PHB is biodegradable; therefore, its usage as an alternative to conventional plastics can help in mitigating the severe environmental impacts of fossil fuel overconsumption as well as the non-biodegradability of plastics. Thus, cyanobacteria such as *Anabaena, Synechocystis, Nostoc muscorum, Spirulina* and many other species can act as biofactories for bioplastic and biofuel production (Kiran et al., 2014).

### Cyanobacteria as Source of Renewable Energy: Tremendous Potential

Bioenergy is a form of energy that is obtained biologically from biomass. Biofuel (either bioethanol or biodiesel) is the only substitute source of energy that can currently replace the transportation fuel in automobiles without requiring extensive engine changes (Kaygusuz, 2009). Biofuel research must include not only the determination of the best material to utilize and its conversion to biofuel but also the ecologically safe and economical use of by-products created during the processing and production of these biofuels in a sustainable manner (Parmar et al., 2011). Development of alternative energy fuel sources is urgently needed, as majority of the world's current fuel supply is reliant on fossil fuels. One of the most challenging problems is the creation of sustainable and clean energy supplies for the future, which is ultimately linked to economic success and stability, as well as higher standards of living on a global scale (Posten and Schaub, 2009). The massive usage of non-renewable fuel sources to meet energy requirements has prompted researchers to look into alternative energy sources (Misra et al., 2013). As a result, biofuels have gained a bigger share of the fuel industry, and their future potential will pave the way for energy security.

**Figure 3:**
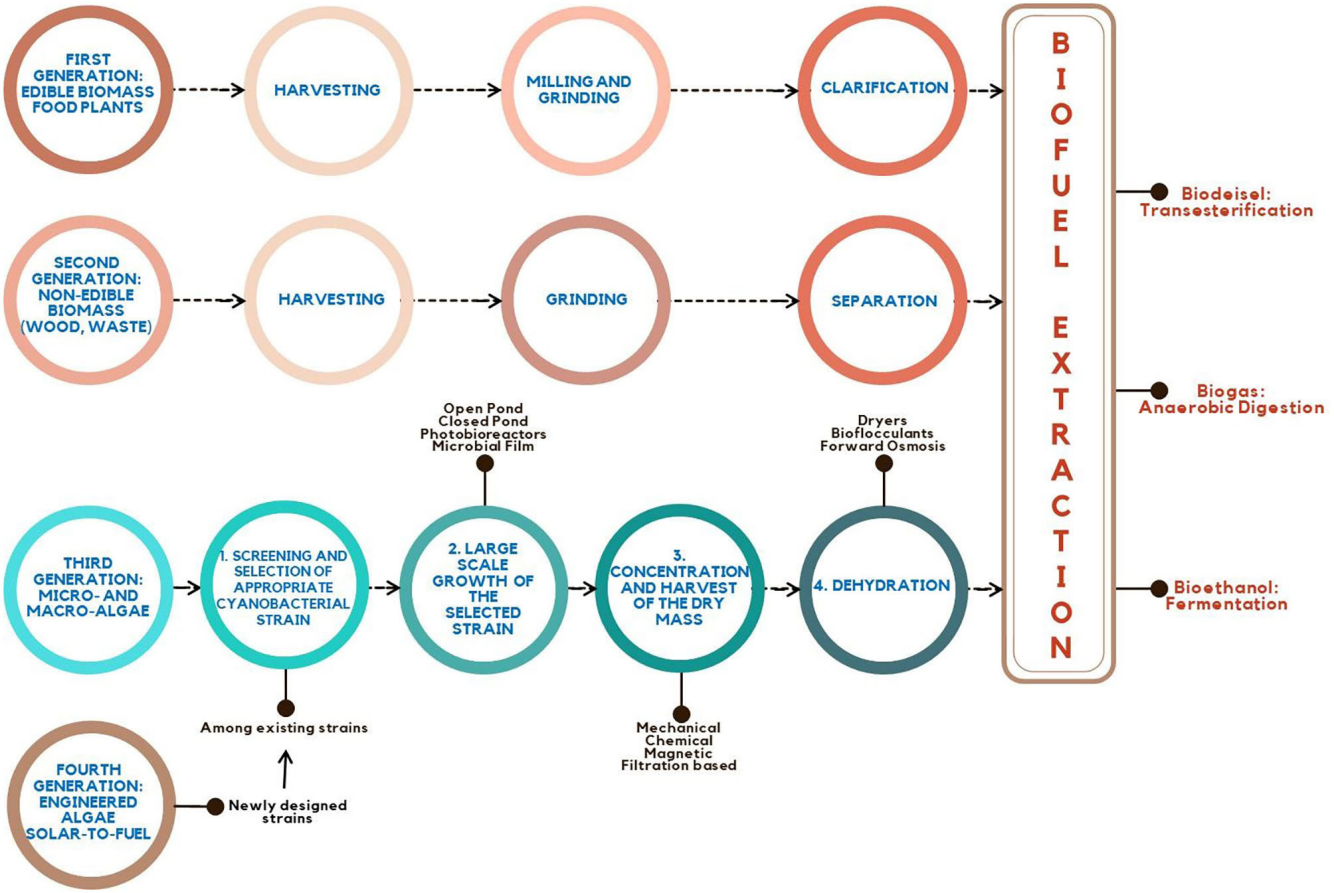
From the first generation to the fourth generation: overview of steps involved in biofuel production technology.

Sunflower, rapeseed, switchgrass, peanuts, wheat, soybean, and sesame are among the most popularly utilized feedstock for the first and second generations of bioenergy. Vegetable oil and alcohols (ethanol, butanol, and propanol) are among the liquid forms of energy generated from these sources. As previously stated, the primary restriction for these energy crops is the competition for acreage and water with our food sources. To alleviate this restriction, third-generation sources are considered as a non-food biomass origin for energy supply (Quintana et al., 2011) (Figure 3).

Cyanobacteria can transform up to 10% of solar energy into biomass, in comparison to the 5% by eukaryotic algae and the 1% by traditional energy crops like sugarcane and maize (Parmar et al., 2011). They also convert CO_2_ into biological components that may be used to make foods, biofuels, feedstock, and highly valued biologically active substances throughout this process (Pereira et al., 2011). Cyanobacteria are viewed as a possible feedstock for carbon-neutral biofuels owing to their high biomass production, quick growing capacity, and ability to manufacture and accumulate considerable quantities (~20–50% of dry weight) of neutral lipids deposited in lipid bodies present in the cytosol (Halim et al., 2013). Overall, cyanobacteria outperform plants and algae in terms of photosynthetic efficiency, although algae produce far more oil. Cyanobacterial species *viz. Synechococcus elongatus* PCC 7942 and *Synechocystis* sp. PCC 6803 are widely utilized and identified as a potential biofuel source. As a result, photosynthetic microorganisms like cyanobacteria can better serve the goal of bioenergy generation in a more cost-effective and ecologically friendly manner, potentially replacing a large amount of fossil fuel consumption (Farrokh et al., 2019). They have a high amount of lipids, mostly found in thylakoid membranes. In comparison to higher plants and other algae, they have higher photosynthetic output and growth rates. Cyanobacteria may grow readily if given only the most basic dietary requirements: air, water, nitrogen, carbon dioxide, and mineral salts (particularly salts having phosphorous) with photons of light as the sole source of energy. As a result, cultivation is straightforward and economical (Quintana et al., 2011). Cyanobacteria have a comparatively short genome, and many of them have already been entirely sequenced, making system biology methods less difficult in these species than in eukaryotic algae (Rittmann, 2008). Cyanobacteria have been genetically modified to create a variety of substances related to biofuel (Machado and Atsumi, 2012; Table 1). *Synechococcus elongatus* sp. strain PCC 7942 was effectively designed to make ethanol by adding a gene for enzymes, alcohol dehydrogenase, and pyruvate decarboxylase, diverting carbon from pyruvate to ethanol (Deng and Coleman, 1999).

**Table 1:** Different cyanobacterial strains genetically engineered for biofuel production.

**S. No**.	**Cyanobacterial strain**	**Organic Carbon utilization**	**Cultivation conditions**	**Specific growth rate**	**Biofuel/biofuel precursor**	**Product concentration**	**Engineered status**	**Reference**
1.	*Cyanothece* sp. ATCC 51142	Oxygenic photo-autotrophic	Chemostat culture-ASP-2 medium, Nitrogen deprivation, Continuous white light illumination	Variable	Hydrogen	400 mmol H_2_/mg Chl. H	Disruption of uptake *hydrogenase* gene	Masukawa et al., 2002
2.	*Synechococcus elongatus* 7942	Photoautotrophic	Modified BG-11 Medium	0.161 day^−1^	Iso-butanol	450 mg/L	Overexpression of ribulose 1,5-bisphosphate carboxylase/oxygenase (Rubisco)	Atsumi et al., 2009
3.	*Synechocystis* sp. PCC 6803	Photo-auto/heterotrophic	BG11 medium	1.7~2.5 day^−1^	Ethanol	5.5 g/L	*pdc-adh* genes set expressed under *PpsbA2* promoter	Dexter and Fu, 2009
4.	*Synechocystis* sp. PCC 6803	-	Dark, Nitrogen limiting	1.7~2.5 day^−1^	Hydrogen	186 nmol/mg chl a/h	*nitrate reductase (ΔnarB)*, *nitrite reductase (ΔnirA)*	Baebprasert et al., 2011
5.	*Synechococcus elongatus* 7942	Photoautotrophic	Modified BG-11 medium	0.161 day^−1^	1-butanol	30 mg/L	substitution of bifunctional aldehyde/alcohol dehydrogenase (*AdhE2*) with separate butyraldehyde dehydrogenase (*Bldh*) and NADPH-dependent alcohol dehydrogenase (*YqhD*)	Lan and Liao, 2012
6.	*Synechococcus elongatus* 7942	Photoautotrophic	BG-11 medium	0.161 day^−1^	2,3 butanediol	2.4 g/L	Integrated *alsS, alsD*, and *adh*	Oliver et al., 2013
7.	*Synechocystis* sp. PCC 6803	Mixoautotrophic	BG11 medium	1.7~2.5 day^−1^	Isobutanol	114 mg/L	*kivd* and *adhA* gene set of Ehrlich pathway expressed under P_tac_	Varman et al., 2013
8.	*Synechococcus* sp. PCC 7002	Photoautotrophic	Agar plates of medium A^+^	0.2 h^−1^	Fatty acids	130 mg/L	*fadD* gene knockout, overexpression of *tesA* and *rbcLS*	Ruffing, 2014
9.	*Synechocystis* sp. PCC 6803	Photoautotrophic	BG11 medium	1.7~2.5 day^−1^	Fatty alcohol for biodiesel	761 μg /g dry cell weight	Overexpression of fatty *acyl-CoA reductase* gene and disruption of the native glycogen/poly-β-hydroxybutyrate biosynthesis pathways	Qi et al., 2013
10.	*Synechococcus* sp. PCC 7002	Photolitho-autotrophic	CO_2_ enriched seawater medium	0.2 h^−1^	Ethanol	0.25% (v/v)	Pyruvate decaroxylase (PDC) gene from *Zymomonas mobilis* and Alcohol Dehydrogenase (ADH) gene from *Synechocystis* 6803	Kopka et al., 2017
11.	*Synechococcus* sp. PCC 11901	Photoautotrophic	MAD, MAD2 medium.	≈100 mgDW h^−1^	Fatty acids	≈1.54 g L^−1^	*tesA* under P_clac143_ promoter, *fadD* knockout	Włodarczyk et al., 2020
12.	*Synechocystis* sp. PCC 6803	Photoautotrophic	BG11 medium	1.7~2.5 day^−1^	3-Methyl 1- Butanol	75 mg/L	Keto-acid Decarboxylase *(kdc)-* Alcohol Dehydrogenase *(adh)* gene set expressed under *CcaS/CcaR* system	Kobayashi et al., 2022

Cyanobacteria are an excellent alternative for producing solar-powered biofuels from water, sunlight, and CO_2_. However, several difficulties for biofuel generation and development in cyanobacteria are investigated by extending natural biosynthetic pathways. Several initiatives have been undertaken to increase biofuel production by expanding natural biochemical routes, including hydrogen, fatty acids, ethanol, isoprene, and butanol. Currently, metabolic engineering techniques and synthetic biology are employed in cyanobacteria to produce high-level biofuels. Different types of biofuels obtained from cyanobacteria are discussed below.

#### Ethanol

Glucose and sucrose are two of the major sugar moieties easily obtained from cyanobacteria. Anaerobic fermentation of these simple sugars in the dark produces ethanol or ethyl alcohol. It is a colorless, flammable, and volatile liquid created by microbes by sugar fermentation. The ethanol made from renewable sources is a desirable source of energy, since it can be combined with diesel and utilized without requiring further modifications to present diesel engines (Kaygusuz, 2009). In the production of ethanol, cyanobacteria have an advantage over conventional energy crops, since they ferment naturally without any need for yeast cultures (fermentation initiators) that are needed by the latter, therefore making cyanobacteria better candidates for ethanol generation. Most algae and cyanobacteria do not need fermentation as their major source of energy. Fermentation occurs at a low level in these species, thus helping them to survive. Genetic modification may be a viable option for overcoming this difficulty and increasing ethanol output from engineered cyanobacteria that are a reliable source for ethanol production (Quintana et al., 2011). *Synechococcus* sp. PCC 7942 was the first genetically engineered cyanobacterial species utilized to produce ethanol. Coding sequences of *Zymomonas mobilis* pyruvate decarboxylase (*PDC*) and alcohol dehydrogenase II (*ADH*) were cloned in the pCB4 vector and utilized to transform *Synechococcus* sp. strain PCC 7942. The promoter from the *rbcLS* operon, which encodes cyanobacterial ribulose-1,5-bisphosphate carboxylase/oxygenase, increased the expression of the *PDC* and *ADH* genes (Deng and Coleman, 1999). Modifying various abiotic parameters like light intensity, carbon source, CO_2_ concentration, pH, and medium composition can boost the production of certain molecules (Gao et al., 2012). In many situations, optimizing these abiotic and biotic elements together has led to increased yields (Andrews et al., 2021). Carrieri et al. (2010) evaluated the influence of salt stress on cyanobacterial fermentation rate. High salt concentration in a medium led to a 100-fold increase in ethanol production as compared to low salt concentration. The amount of ethanol released by cyanobacteria has a significant impact on two parameters, the amount of energy used and GHG emission. Cyanobacteria derived ethanol do not produce any remnant, ash, smoke and greenhouse gases in any form therefore serves as an eco-friendly fuel (Luo et al., 2010). Multiple ethanologenic cassette insertions inside the *Synechocystis* genome have been shown to be a successful technique for increasing ethanol titers (Gundolf et al., 2019; Wang et al., 2020). However, in cyanobacteria, plasmid-based ethanol-generating systems have been highly effective and have frequently resulted in rather large ethanol titers (Dühring et al., 2017). Using a plasmid-based system, the patented strain ABICyanol1 has attained an ethanol output of 0.55 g/L/d (Piven et al., 2015). According to an *in silico* study, the maximal amount of ethanol produced by *Synechocystis* sp. PCC 6803 was found to be 235 percent more than what was produced experimentally on the laboratory scale. The researchers were able to do this by developing a combined ethanol/biomass system (Lasry Testa et al., 2019). Cyanobacteria have the potential to produce lipids, which may be utilized as biofuel feedstock (Anahas and Muralitharan, 2018). Recently, Roussou et al. (2021) investigated the combined effects of Calvin-Benson-Bassham (CBB) enzymes like transketolase (TK), aldolase (FBA), and fructose-1,6/sedoheptulose-1,7-bisphosphatase (FBP/SBPase) on ethanol production in *Synechocystis* PCC 6803. The strain that overexpressed a combination of FBA and TK CBB enzymes produced the maximum amount of ethanol.

#### Butanol

Butanol (butyl alcohol) is a 4-carbon alcohol (C_4_H_9_OH) that forms the main bulk chemical and an effective blend-in fuel sourced from non-renewable fossil resources (Liu et al., 2021). 1-Butanol is gaining interest as a potential fuel alternative and an important chemical feedstock. It can be produced by both the coenzyme A (CoA)-dependent route (Ezeji et al., 2007) and the ketoacid pathway (Shen and Liao, 2008) (Figure 2). There are also biological pathways for fermentative butanol synthesis (Wichmann et al., 2021). Natural cyanobacterial strains do not synthesize isobutanol or 1-butanol, suggesting that butanol metabolic genes and related pathways are missing. For heterotrophic 1-butanol synthesis, the clostridial route, a natural 1-butanol-generating pathway from *Clostridium*, was introduced into *E. coli* (Shen et al., 2011). In *Synechococcus* 7942, Atsumi et al. (2008) created a synthetic isobutanol pathway that generates isobutyraldehyde and isobutanol. Similarly, an alternative acetoacetyl-CoA biosynthesis pathway (ATP-driven) has been created in *Synechococcus* by overexpression of acetoacetyl-CoA synthase (*NphT7*) that condenses malonyl-CoA and acetyl CoA. In *Synechococcus*, the overexpression of three enzymes (phosphoglycerate mutase, enolase, and pyruvate kinase) from the three stages between 3-phosphoglycerate and the pyruvate of the Calvin–Benson–Bassham cycle boosted overall carbon output by 1.8-fold and 2,3-butanediol synthesis by 2.4-fold (Oliver and Atsumi, 2015). Miao et al. (2018) reported a maximum 1-butanol and isobutanol production in *Synechocystis* PCC 6803 during a long-lasting culture (0.9 g/L in 46 days). CRISPR interference (CRISPRi) was conducted in another investigation to repress the enzyme citrate synthase gltA, which arrests growth but increases carbon partitioning leading to the production of 1-butanol in *Synechocystis* (Shabestary et al., 2018).

Furthermore, the synthesis of 1-butanol was boosted by overexpressing a heterogeneous phosphoketolase (PK) route, one of the natural acetyl-CoA supporting routes in *Synechocystis* (Xiong et al., 2015; Liu et al., 2019). Wichmann et al. (2021) recently showed that photosynthetic butanol is created directly from CO_2_, with a carbon partitioning of 60% to 1-butanol.

#### Biodiesel

Biodiesel, unlike petroleum diesel, is biodegradable and non-toxic, and when burnt as a fuel, it considerably decreases toxicants and other emissions (Yusuf et al., 2011). Cyanobacteria are photosynthetic microorganisms that can convert solar energy and CO_2_ into biofuels in a single biological system. Synthesizing fatty acid-based compounds using solar energy as the energy source, CO_2_ as the carbon source, and cyanobacteria as the biological system would be a potential technique for developing sustainable biofuels (Quintana et al., 2011). In combined liquid–gaseous fuel-processing solutions, S*pirulina* also demonstrated the greatest overall utilization efficiency. In cyanobacteria, overproduction and transesterification of fatty acids to generate fatty acid methyl esters, fatty alcohols, or fatty alkanes are the two processes involved in the production of fatty acid-based biofuels (Mata et al., 2010). These lipid feedstocks are made up of 90–98 percent triglycerides and trace quantities of monoglycerides and diglycerides, 1–5 percent free fatty acids, and small amounts of phosphatides, phospholipids, carotenes, sulfur compounds, tocopherols, and water (Chisti, 2007). Because of their high fatty acid content, Hossain et al. (2020) discovered that cyanobacteria represent a promising source for the biodiesel sector. Cyanobacteria have high lipid content, with Oscillatoriales having the greatest overall lipid concentration. More studies are needed to improve the mass culture conditions for increasing the lipid content of cyanobacterial biomass, and research on value addition of residual biomass is also required. Nagappan et al. (2020) evaluated numerous nitrogen-fixing cyanobacteria for biodiesel production based on biomass production, lipid productivity, lipid profile, and harvesting capability. The evaluation resulted in the selection of *Nostoc* sp. MCC41, a nitrogen-fixing cyanobacterium and a promising species. It contains a high proportion of palmitic acid, indicating its applicability for biodiesel production. *Nostoc* sp. *MCC41* can be regarded as a promising and eco-friendly resource for efficient biodiesel production because of its ease of harvesting, capacity to grow under mixotrophic conditions, and ability to fix atmospheric nitrogen. To investigate the activity of certain enzymes used in saturated fatty acid production in cyanobacteria, genetically altered *Synechococcus elongatus* PCC 7942 was generated by overexpression or deletion of genes coding for fatty acid synthase system enzymes; its lipid profile was evaluated, and it was found that the functioning of some of these enzymes differed. Modifications resulting from gene overexpression or deletion were then conducted to increase the production of alpha-linolenic acid in cyanobacterium. When combined with the overexpression of *Synechococcus* sp. *desA* and *desB* desaturase genes, the mutant generated by *fabF* overexpression and *fadD* deletion, PCC 7002, generated the most omega-3 fatty acids (Santos-Merino et al., 2018).

#### Hydrogen

Because it is non-polluting and infinite, hydrogen (H_2_) is a more appealing biofuel candidate for upcoming usage (Srirangan et al., 2011). According to Kruse et al. (2005), many cyanobacteria spontaneously create hydrogen as a secondary metabolite that balances redox energetics, and many other strains can create hydrogen by the reversible action of hydrogenase. The most significant impediment to cyanobacterial H_2_ production is that hydrogenases are particularly sensitive to the O_2_ produced during photosynthesis. Furthermore, the presence of reducing agents like ferredoxin and NADPH is a barrier, since they are involved in other processes such as respiration. To boost hydrogen generation, it will be necessary to reroute some of the electron flow to hydrogen-producing enzymes and to develop hydrogenases tolerant to oxygen (Weyman, 2010). When cyanobacteria are cultured under nitrogen-deficient conditions, H_2_ evolves as a by-product of nitrogen fixation. It was also discovered that non-heterocystous cyanobacteria produce lesser hydrogen than heterocystous species. Several studies have looked at cyanobacterial species that can produce H_2_ as a source of clean biofuel (Abed et al., 2009). Multiple attempts have been made to boost H_2_ production rather than carbon flow to augment electron flux. Nitrogenase-based generation systems of H_2_ have been created in *Nostoc* sp. PCC 7120 by inhibiting hydrogenase (Hup) absorption, resulting in increased hydrogen (Masukawa et al., 2002). *Synechococcus* 7002 mutants deficient in lactate dehydrogenase showed a 5-fold increase in total H_2_ generation when compared with the wild type (McNeely et al., 2010). The production of an exogenous ferredoxin by *Clostridium acetobutylicum* during the fermentation process might boost electron flow to the hydrogenase (HydA). In light-dependent anoxic circumstances, H_2_ production was increased twice its original amount (Ducat et al., 2011). Several initiatives have been launched to hinder competitive pathways for reductant usage and to promote H_2_ production. As a consequence, the *ldhA* gene was inactivated in *Synechococcus* sp. 7002, NADH/NAD ratios were significantly increased, and H_2_ production was significantly enhanced when native bidirectional [*NiFe*] hydrogenase was increased (five-fold in anoxic and dark circumstances) (McNeely et al., 2010). Engineered *Anabaena variabilis* ATCC 29413, *Nostoc* sp. PCC 7422, and *Nostoc linckia* HA-46 generated more H_2_ than *Synechococcus* sp. PCC 7002, *Synechocystis* sp. PCC 6803, and *Nostoc punctiforme* ATCC 29133. In contrast, the lowest H_2_ producers have been found to be *Synechococcus elongatus* PCC 7942, *Synechococcus* sp. PCC 7002, and *Synechocystis* sp. PCC 6803d. It implies that during H_2_ synthesis, oxygen may function as an inhibitor in these cyanobacteria. As a consequence, an artificial oxygen-consuming device for O_2_ consumption may be built in the near future, resulting in enhanced H_2_ production in *Synechocystis* sp. PCC 6803 and *Synechococcus* sp. PCC 7002 (Srirangan et al., 2011). Khetkorn et al. (2012) used multiple inhibitors to promote electron flow toward nitrogenase and bidirectional Hox-hydrogenase in *Anabaena siamensis* TISTR 8012 to study the downregulation of those competing for metabolic pathways. Increased H_2_ generation produced by inhibitors corresponded to increase in respective Hox-hydrogenase activity and nif D transcript levels and decrease in hupL transcript levels. The NiFe-hydrogenase HoxYH from the cyanobacterium *Synechocstis* sp. PCC 6803 was coupled with the photosystem I component PsaD. The resultant psaD-hoxYH mutant grew photoautotrophically, accumulated a large concentration of photosynthetically produced hydrogen in the light under anaerobic conditions, and did not consume hydrogen. According to the findings, psaD-hoxYH photosynthetic hydrogen production is most likely a mix of anoxygenic and oxygenic photosynthesis (Appel et al., 2020).

#### Conversion and Downstream Processing for Large-Scale Production of Eco-Friendly Cyanobacterial Biofuels

##### Steps of Conversion of Cyanobacterial Biomass to Biofuels

The scientific community is continuously looking for alternatives and optimizations to improvise and achieve an efficient, practical, and economically viable bioenergy process (Figure 3). However, the general pathway is as follows:

*Selection and Screening of Cyanobacterial Strains With Desirable Fatty Acid Profiles.* It will determine the quality of the biofuel produced eventually. Not only selection from among the existing cyanobacterial strains but many promising strains designed by genome modeling strategies have also been developed, especially in a popular cyanobacterium, *Synechocystis* sp. PCC 6803 (Erdrich et al., 2014), to yield an economically feasible level of biofuel.

*Large-Scale Growth of Cyanobacterial Strain.* Multiple approaches have been adopted for achieving high biomass productivity (Jorquera et al., 2010) viz: (a) Open pond systems where open waters with nutrients are supplied for algal growth. However, such systems are prone to bacterial and protest contamination. (b) Closed pond systems that utilize closed chambers to provide round-the-year cultivation owing to full control over light and temperature conditions. (c) Photobioreactors that are high on investment and need technology optimization but tend to maximize the photosynthetic surface area. (d) Microbial biofilm method wherein the microbial paste is applied on a suitable substratum to directly produce the biomass, thereby omitting the harvesting step.

A few prerequisites must be fulfilled to achieve viable biomass and include sufficient nutrient inputs, maintenance of sterile conditions to prevent contamination from other microbes, and optimization and maintenance of strain-conducive environmental conditions of light and temperature (Sitther et al., 2020).

*Harvest/Collection Through Concentration and Dry Mass Production.* Strategies including batch centrifugation (mechanical separation method), flocculation (inorganic vs. organic separation methods), use of magnetic nanoparticles, reverse and/or direct vacuuming (filtration-based method), and flotation (natural formation of gas vesicles) have been constantly employed for harvesting biomass (Parmar et al., 2011; Sitther et al., 2020).

*Drying/Dewatering/Dehydration.* Dehydration is carried out to prepare the biomass for extraction of biofuel using huge drums with oven dryers. This dewatering process can be performed either by addition of bioflocculants (usually bacterial cells) in the cyanobacterial culture, which saves the additional costs of chemical flocculants, but increases the chances of contamination and interference with cyanobacterial metabolic processes, or another efficient technology of forwarding osmosis could be employed that works on creating a pressure gradient across a semi-permeable membrane. This method is highly efficient and can recover cyanobacterial cells from dilute cultures as well (Anyanwu et al., 2018).

*Extraction and Purification.* Extraction and purification of third-generation biofuels are accomplished using multiple techniques *viz*:

a. Transesterification for biodiesel production is the most conventional process for optimized generation of lipid-based biofuel. In this process, fatty acids (triglycerides) and methanol are converted into glycerol and FAMEs (fatty acid methyl esters), in the presence of a strong alkali or a strong acid (Bhatia et al., 2021). Conventional 2-step transesterification, which involves sequential lysis and transesterification, does not yield ample FAMEs from cyanobacteria; therefore, a highly efficient single-step method has been developed where cell lysis and transesterification of fatty acids occur simultaneously, which can be chemically separated by phase separation. This direct transesterification (also called *in situ* transesterification) allows for quantification and characterization of fatty acids without involving a separate method of extraction (Wahlen et al., 2011).

b. Fermentation for bioethanol production: cellular fermentable sugars and polysaccharide glycogen are present in cyanobacteria that can be optionally enzymatically hydrolyzed and converted to ethanol and carbon dioxide in the complete absence of oxygen (Lakatos et al., 2019).

c. Anaerobic digestion for biogas production: various conversion pathways have been standardized to obtain gas-based cyanobacterial fuels where cyanobacterial residual biomass (post-liquid fuel extraction or direct from culture/sludge) can be anaerobically digested by hydrolysis (conversion of initial biomolecules into soluble sugars), fermentation (sugar conversion to alcohol and intermediary biomolecules), and methanogenesis (conversion to biogas mixture comprising up to 70 percent methane using methanogens) (Fardinpoor et al., 2021). Cyanobacterial biohydrogen is produced under nitrogen-deficient conditions because of the reverse activity of enzyme hydrogenase. Some strains of *Anabaena* spp. are known to have a maximum potential to produce the highest amounts of biohydrogen (Sadvakasova et al., 2020).

*Analysis, Testing for Marketability and Approvals for Commercialization of the Biofuel Product: In Terms of Rate of Titer Production, Its Quality, and Steadiness.* International standards and specifications are continually being introduced to harmonize the quality and testing methodologies of biofuel products globally (Gadonneix et al., 2010). ISO (International Organization for Standardization) keeps developing and updating these standards to help in efficient development and global acceptance of biofuel products.

##### Light-Mediated (Direct) Use of Cyanobacteria as Biofuels

Cyanobacteria have an upper hand over other biofuel candidates because of their inbuilt efficiency to utilize sunlight and convert biomass into fuels. Light-driven conversion works smoothly without any additional processing steps. Therefore, by employing multiple interdisciplinary approaches, we can alter and improvise metabolic pathways directly achieving high yields of the fourth-generation drop-in biofuels **(**Johnson et al., 2016). The basic raw materials (carbon dioxide, water, and light) are provided, and the modified genetic makeup leads to the direct production of high-value biofuel derivatives such as ethanol and butanol. This is a rapidly growing field of study where new ways of altering the genetic makeup of cyanobacterial strains can be conducted resulting in not only improved yields but also single step biofuel production. *Synechococcus* sp. PCC 7002, with overexpression of native sodium-dependent bicarbonate transporters SbtA and BicA, yielded a 50% increase in growth/biomass and intracellular glycogen yield (Gupta et al., 2020). Fan et al. (2022) worked on *Synechocystis* sp. PCC 6803 and studied variations in the efficiency of substrate conversion into aromatic alcohol by optimizing permutations and combinations of parameters like temperature, light, substrates, and cell concentrations. They successfully achieved higher NADPH regeneration and, hence, improved reaction rates in terms of alcohol production.

The advent of fourth-generation biofuels from inexhaustible raw material sources like cyanobacteria, which are cheap and easily available, has opened new hopes. A photosynthetic mechanism involving water oxidation can lead to large-scale fuel production either by an artificial photosynthetic process (Inganäs and Sundström, 2016) or by directly opting for methods of solar biofuel production that is a much cleaner source of energy. This will not just take up hydrogen production but will certainly minimize the generation of reduced carbon-based biofuels by concomitant atmospheric CO_2_ fixation and by designing synthetic pathways for enhanced biofuel outputs (Mund et al., 2022; Sebesta et al., 2022). An approach toward designer cyanobacterial strains with edited metabolic pathways can troubleshoot many environmental concerns at the industrial level for biofuel production. The biggest challenge to be resolved is identifying controlled expression systems in designer cyanobacteria. To sum up, the fourth generation biofuels, which are a by-product of the designer microbes including cyanobacteria, are utilized as photobiological solar fuels designed to work in coalition with photovoltaics or electro biofuels. Cyanobacterial strains having their metabolism tailored specifically for enhanced biofuel production is going to take up the mere future. Additionally, using computational modeling methods for identification of the complex interplay of potential pathways, the flux can be manipulated in order to achieve higher and efficient biofuel yields (Misra et al., 2013).

### Bioplastics: Bio-Based, Biodegradable, and Eco-Friendly Plastics

Plastics are primarily composed of polymers and include a wide range of semisynthetic and synthetic materials. Moreover, because of plasticity, plastic objects can be remolded into a wide range of solid shapes by pressurizing them. Fossil fuels such as natural gas and petroleum are used to make plastics recently; however, industrial methods use renewable materials, namely, corn or cotton derivatives. Flexibility and other properties like being lightweight, durability, and low production cost have led to their extensive use worldwide. Plastic objects, having lower weight and cost, have an additional advantage and wide applications in industries and markets (ACC, 2013; Lane, 2015). In industries, plastics are used to meet numerous requirements that are very specific in having a high strength-to-density ratio. In plastics, polymers have a high degree of chain branching and cross-linking that makes them rigid; and for transparency, polymers with variable glass transition temperatures are used. Different polymers can be blended to make products with desired properties. Therefore, depending on density and high resistance under different ambient suitable conditions, the most widely produced thermoplastics that have multiple uses in industries are polyethylene terephthalate (PET), low-density polyethylene (LDPE) and high-density polyethylene (HDPE), polyvinyl chloride (PVC), polypropylene (PP), and polystyrene (PS) (ACC, 2007). These properties and ability to be remolded provide long shelf life to numerous plastic products; therefore, plastic polymers can be used in packaging, storing, and transportation of materials even to long distances. Production of plastic polymers is increasing worldwide and was reported to be more than 300 million tons in the past few years (Plastics, 2019). Conventional petrochemical-based plastics impose serious environmental hazards. On the contrary, unrestrained usage of non-durable goods made from plastic polymers generated a vast amount of uncalculated waste in the environment. It also imposes a severe threat to ecosystems because of their slow decomposition process in the natural ecosystem, and it is one of the global concerns that the world needs to address. However, plastic polymers are non-biodegradable, they rely on the population boom and demand. As minimum plastic waste is known to be recycled therefore, reducing the demand at an individual level, lowering human pressure on environment and creating interest to develop the better disposal mechanisms can be the ways for sustainable future.

Now, bioplastics are a better-known option, sustainable, and a suitable alternative to conventional synthetic chemical-based plastics and are well-recommended by the United Nations (UN) recently. Because of environmental safety concerns, many agencies including the UN Food and Agriculture Organization (FAO) assessed the sustainability of bio-based plastic materials and strongly recommended bio-based polymers that can be biologically degraded over conventional non-biodegradable polymers. Plant-based biodegradable bioplastics can be an alternative sustainable source of plastics. However, land requirement for proper plant growth can be a limitation in developing nations where no well-equipped land use pattern is followed (European Bioplastics, 2021).

Bioplastics (bio-based plastics) are synthesized at least partially from biological matters as well as plastics that can be biologically degraded. Biodegradable or biologically degradable plastics are broken down or completely degraded or decomposed by microbes under particular conditions in due space within a certain period. As observed and based on composition, it is a very interesting fact that every plastic product that is bio-based cannot be biodegradable, and all biodegradable plastics products cannot always be bio-based. Moreover, under all environmental conditions, biodegradable plastics cannot be degraded biologically. Renewable resources used for obtaining bioplastics, are recycled *via* varied natural biological phenomena and can help in reducing fossil fuel consumption (Ashter, 2016).

The potential studies conducted by “Organization for Economic Co-operation and Development” (OECD) further provided many definitions for plastic polymers such as bioplastics are bio-based, and while their production generates very less and cleaner residues, their decomposition mechanisms are less detrimental than conventional chemical-based plastics to the environment (Reis et al., 2003; Schlebusch and Forchhammer, 2010; Jim, 2014). Despite of their origin, bioplastics can also be referred based on how they can be degraded by different organisms namely, bacteria, fungi and algae (Rutkowska et al., 2002). Polysaccharides, namely, cellulose and starch, and various polyesters like polyhydroxyalkanoates (PHAs) are bioplastics that can be the potential and most promising source of environmentally safe polymers (Storz and Vorlop, 2013).

Until now, bio-based and non-biodegradable plastics are being manufactured in the market; however, biodegradable polymers are not leading; therefore, creating biopolymers with high performance and reasonable cost is a matter of highest environmental concern (Iles and Martin, 2013). Consequently, because of an urgent need to find an alternative to conventional plastics, and likely PHAs and PHBs, bio-based and biodegradable polymers will require high production capacities at considerate rates in the near future (Aeschelmann et al., 2016). As to growing environmental safety concerns, industries are more attracted to biodegradable plastics that are short-lived and that can be suitably disposed of. In the degradation of plastic polymers, various microorganisms decompose the polymers in the following order as PHAs = PCL (polycaprolactone) > PBS (polybutylene succinate) > PLA (polylactic acid) (Tokiwa et al., 2009; De Paula et al., 2018). Among the plastic polymers, PCL is fossil-based; however, PBS is not fully bio-based, and PLA has gained a growing amount of interest (Lackner, 2015). However, the production capacity of PHAs is still small but is speculated to grow exponentially in the near future (Aeschelmann et al., 2016).

Among PHAs, PHBs are widely studied and comprise the most common PHA. To date, PHBs are the only widespread PHAs produced under photo-autotrophic conditions (Troschl et al., 2018). Polymers such as PHAs, and especially PHBs, have physicochemical characteristics similar to those of petrochemical plastic and can have wide applications. PHBs are crystalline and are comparatively rigid having a methyl group as a side chain. PHBs decompose at ~200°C, i.e., near their melting temperature, show poor melt stability, and turn brittle within a few days of manufacturing. Blending and incorporating other co-monomers can change the chemical properties and other characteristics like decreasing the aging process and, hence, increasing their applications widely. PHBs display less resistance to solvents and high natural resistance against photodegradation (Lane, 2015; Gomes Gradíssimo et al., 2020). Therefore, it is an emergent need to use microorganisms such as cyanobacteria that have low nutritional requirements to produce useful, environmentally safe, bio-based, and biodegradable PHAs.

#### PHA and PHB Structure

**Figure 4:**
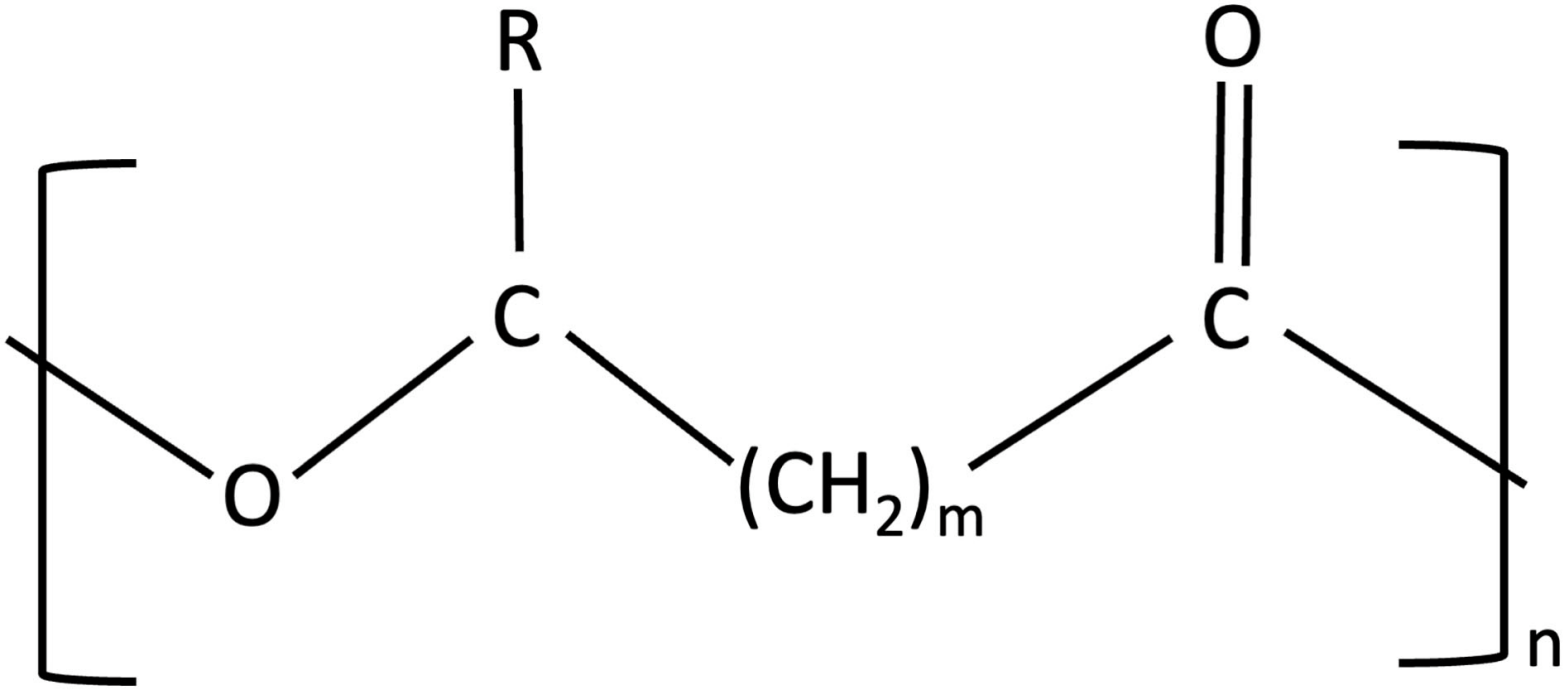
Structure of PHAs (polyhydroxyalkanoates). In homopolymers, m can vary from 1 to 3 {polyhydroxybutyrate (PHB) - m = 1}, n can vary from 100 to 30,000 monomers, where R is a varied chain length alkyl group.

PHAs are linear polyesters having 3–6 hydroxy acids and more than 150 monomers accounting for around 2 million Daltons (Figure 4) (Chen and Wu, 2005; Gomes Gradíssimo et al., 2020). Alcohol, sugar, and alkane production can act as a substrate for the formation of polymers. The varied physical properties are due to the different chemical structures of PHAs produced from different bacterial genera becoming more suitable for wide applications (Raza et al., 2018). PHAs, however, are one of the largest categories of natural bio-based polyesters composed of more than 150 different monomers of PHAs (Pittman et al., 2011). Varied changes in structural configurations and variations of monomers can form homopolymers of PHAs and copolymers of PHAs, and an example of a homopolymer is PHB and a copolymer is poly(3-hydroxybutyrate-co-3-hydroxyvalerate) (PHVB) (Chen, 2009). Based on polymerization, PHAs can be of different lengths such as short-chain length (SCL) including PHB, poly(3-hydroxyvalerate)-PHV, and their copolymer PHBV, while another type of PHAs is medium chain length (MCL) that includes poly(3-hydroxynonanoate)-PHN and poly(3-hydroxyoctanoate)-PHO.

#### Properties of PHAs

Cyanobacterial PHAs exhibit thermostability and physical properties like those of conventional plastics derived from fossil origin (Gadgil et al., 2017). The physical and chemical properties are determined by their chemical structure, i.e., the number of monomers. The shorter PHA is brittle and shows high crystallinity, while polymers with more number of monomers are flexible and elastic. It covers biopolymers that are biodegradable as well as biocompatible (Chen and Wu, 2005; Tokiwa et al., 2009; Tan et al., 2014; Gomes Gradíssimo et al., 2020). The decomposition of PHAs under natural conditions or by bacterial action depends on various factors like polymer composition, humidity, and temperature of the surrounding. It also depends on the type of decomposing microorganism, as it affects the duration of degradation because different bacteria express diverse types of PHA-depolymerase that degrades the biopolymer. Moreover, the chemical and physical properties of bioplastics result in sinking of these bioplastics in an aquatic environment that facilitates their transformation into carbon and water by decomposers (Tokiwa et al., 2009; Raza et al., 2018).

Interestingly, PHAs like synthetic plastics can be customized and thus are attractive biomaterials, as they can be designed to suit a specific function. Blending PHAs with other biodegradable polymers can considerably improve the physical properties like crystallinity, glass transition temperature, and mechanical properties (Li et al., 2016). Owing to their unique versatility and ecologically friendly biodegradable properties, PHAs are of great use, as they have a wide range of applications and show to be a promising substitute to petrochemical polymers. Usually, petrochemical plastic wastes are either fragmented into microplastics or are being collected into the oceans as garbage patches. However, bioplastics have met the standard specifications for marine degradability. Based on growing needs, PHAs nowadays are most attractive to producers, and the bioplastic market is expected to strongly increase the production capacity of PHAs by many folds in the future (Aeschelmann et al., 2016; Rosenboom et al., 2022). They are widely used in industries to manufacture biodegradable water-resistant surfaces, for controlled pesticide delivery, nanocomposite materials, mulch films, medical devices, tissue scaffolding, plastic packaging, etc. (Philip et al., 2007; Gomes Gradíssimo et al., 2020).

PHAs producing organisms are categorized into two groups: one group that produces PHAs during their growth period and another group that synthesizes PHAs when they grow under limited nutrient conditions (Basnett et al., 2017). Cyanobacteria are the only prokaryotic species known to produce the homopolymer of PHB naturally in photoautotrophic and chemoheterotrophic environments (Rani and Sharma, 2021). The unsteady growth during the fermentation process promotes PHA production. The accumulation depends on the presence of various elements in the growth medium as well as the ionic strength of the medium (Chen, 2009). Recently, cyanobacteria were genetically transformed with PHB and PHA synthesis encoding genes (Noreen et al., 2016).

#### Biosynthetic Pathway for PHA Production in Cyanobacteria

Three biosynthetic pathways are involved in the assimilation of carbon into different polymers. The foremost well-described pathway found in cyanobacteria for PHA production, especially PHB, is similar to earlier documented pathways in heterotrophic and other archaebacteria species (Lim et al., 2002; Lee et al., 2005; Singh and Mallick, 2017). Among cyanobacteria, *Synechocystis* PCC6803 is widely used as a model organism for gaining insight into the generation of PHBs. PHB biosynthesis involves majorly three enzyme-mediated steps: condensation, reduction, and polymerization. Step one includes reversible condensation (Claisen type) of the precursor, i.e., two molecules of acetyl-CoA derived from the tricarboxylic acid (TCA) cycle with an enzyme, ketothiolase, and produces acetoacetyl-CoA. The first step is followed by reduction of acetoacetyl-CoA (encoded by gene *phaA*) with NADPH-linked enzyme acetoacetyl-CoA reductase (encoded by *phaB*) and produces d(-)-3-hydroxybutyryl-CoA. Furthermore, in the last step, d (-)-3-hydroxybutyrate-CoA gets polymerized by the PHA-synthase enzyme (encoded by genes *phaC* and *phaE*) and finally produces poly(3-hydroxybutyrate) biopolymer (PHB) (Figure 2). The four genes, slr1993- *phaA*, slr1994- *phaB*, slr1830- *phaC*, and slr1829- *phaE* encoding the three enzymes are involved in PHB synthesis. In anoxygenic purple sulfur bacteria, the PHA synthase coding mediated by genes *phaC* and *phaE* is similar to that in a cyanobacterium (Lane and Benton, 2015). The role of Rubisco in CO_2_ assimilation is additionally observed in the production of PHB involving the conversion of glycolate to 2-phosphoglycolate synthesis under photosynthetic conditions. Along with the conjugation of PHB synthesis, the substrate propionic acid is used in the synthesis of PHBV copolymer (Balaji et al., 2013). Reportedly, different conditions showed different production rates of PHBs. Under photo-autotrophic conditions in *Synechocystis* sp., the production of these biopolymers was up to 20% (DCW, dry cell weight); however, under heterotrophic conditions, acetate supplementation, and phosphorus deprivation, the production was 28.8% (DCW), and when genetically modified organisms were supplemented with acetate, they showed a higher yield of PHB at 35% (DCW). Interestingly, the highest yield of 55% was obtained when *Synechococcus* sp. MA19 was grown with Ca_3_(PO_4_)_2_ (Panda et al., 2006; Khetkorn et al., 2016; Kamravamanesh et al., 2017; Gomes Gradíssimo et al., 2020).

However, another interesting biosynthetic pathway involves metabolism of lipids and transformation of different alkanes, alkenes, and alkanoates to generate MCL-PHA polymers. Furthermore, PHA synthase enzymes polymerize different hydroxyalkanoate monomers during the fatty acid oxidation pathway (Hein et al., 2002; Lim et al., 2002).

In the next and third biosynthetic low production cost involved pathway, MCL-PHAs are polymerized from monomers that are produced from glucose, fructose, and sucrose (Philip et al., 2007). Here, with a glycolic precursor and intermediates of the fatty acid biosynthesis pathway, lipids and carbohydrates are used in the generation of PHAs. PHA generation, accumulation, and growth of microbes are also affected by the availability or deprivation of many nutrients and their concentrations, mainly including nitrogen, phosphate, sulfur, and oxygen. Optimum changes in carbon-to-nitrogen ratio are used for culture optimization, whereas abundant carbon is favorable for the production of biomass, and limiting the concentration of phosphorus and/or nitrogen is found to beneficial for the generation of polymers of PHAs (Salehizadeh and Van Loosdrecht, 2004; Wen et al., 2010; Montiel-Jarillo et al., 2017). In cells, nitrogen is required for manufacturing proteins and nucleic acids, and the availability and deprivation of nitrogen levels influence NAD(P)H concentration, NAD(P)H/NAD(P) ratio, and, hence, the TCA cycle (Lee et al., 2005; Albuquerque et al., 2010; Reddy and Mohan, 2015). In a balanced well-supplemented nutrient culture, the TCA cycle is maintained as the concentration of NAD(P)H, and NAD(P)H/NAD(P) ratio remains constant. On the contrary, in nitrogen deficiency, amino acid synthesis, α-ketoglutarate conversion into glutamate, assimilation of ammonium ions, and NAD(P)H accumulation get affected (Panda et al., 2006; Liu et al., 2011) and, hence, influence PHB production.

In cyanobacteria, other than nitrogen, the availability and deprivation effect of phosphorus are found to be more significant. Despite the role in cell maintenance and lipid and carbohydrate assimilation, mainly the inorganic form of phosphorous is a part of protein and nucleic acid (Reddy and Mohan, 2015; Gomes Gradíssimo et al., 2020). Therefore, in well-balanced culture, nutritional growth conditions with a suitable concentration of phosphorous and high coenzyme-A (CoA-SH) concentration inhibit the synthesis of PHA, whereas during phosphorous deprivation, the Krebs cycle gets restricted (Montiel-Jarillo et al., 2017). The deprivation metabolism of nitrogen and phosphorous promotes the reducing factor, NADH accumulation; citrate synthase concentration decreases and isocitrate dehydrogenase and acetyl-CoA precursor concentration increases and, hence, influence PHA biosynthesis (Singh and Mallick, 2017; Gomes Gradíssimo et al., 2020).

#### Genetic Engineering of Cyanobacteria for Enhanced Bioplastic Production

Cyanobacteria serve as a promising and sustainable substitute to produce biopolymers like PHB and offer several advantages in comparison to heterotrophic bacteria (Khetkorn et al., 2016). Despite various advantages, still, they have not found acceptance in the market as cyanobacterial strains are still not optimized for industrial processes. Genetic engineering is one of the techniques that have been exploited to improve the production of PHBs. There have been numerous research studies on genetic engineering in plants for the production of bioplastics, which are referred to as first- and second-generation bioplastics, but now the third generation that is commonly known as “algal bioplastics” is gaining immense importance as it offers a substitute for sustainable production of bioplastics. Genetic studies on cyanobacteria have a major advantage in terms of genetic manipulation in comparison to plants as their genome is less complex and, thus, is easy to manipulate (Koksharova and Wolk, 2002; Koller, 2020). However, most studies on biosynthesis and metabolic engineering of cyanobacterial PHB have been conducted with restricted strains of cyanobacteria. Out of all, *Synechocystis* sp. PCC 6803 is a largely applied model organism. It was the first phototrophic organism to be completely sequenced with mutant variants readily available and thus is largely used in gene manipulation to improve PHA production. There are a number of reports where it has been efficiently transformed for the production of PHBs and for the synthesis of biohydrogen, isoprene, and other compounds (Kaneko and Tabata, 1997; Kamravamanesh et al., 2017; Yashavanth et al., 2021).

Photosynthetic prokaryotes can be genetically engineered by transformation with genes encoding the enzymes required for PHB biosynthesis like acetoacetyl-CoA reductase, β-ketothiolase, and PHB synthase. The wild type cyanobacterial strains like *Aulosira fertilissima, Nostoc muscorum*, and *Synechocystis* sp. PCC 6803, which exhibit native PHB biosynthesis pathway, can be genetically engineered for achieving high yield and PHB productivity (Bhati et al., 2010). Mobilization of complete operons or genes from some other microorganisms that produce PHBs into the cyanobacteria can also boost PHB biosynthesis. In addition to the genes associated with the PHB pathway, the overexpression or deletion of other genes has been found to amplify the level of acetyl-CoA and PHB (Yashavanth et al., 2021). Takahashi et al. (1998) reported that recombinant *Synechococcus* sp. PCC7942 obtained by transformation with genes from *Alcaligenes eutrophus* that encode poly-3-hydroxybutyrate (PHB)-synthesizing enzymes showed improved PHB content in response to CO_2_ enrichment under photoautotrophic conditions and nitrogen starvation. Moreover, the addition of acetate enhanced PHB content by more than 25% under conditions of nitrogen starvation. Miyake et al. (2000) found that inserting the PHB synthase gene obtained from *Ralstonia eutropha* in *Synechococcus* sp. MA19 resulted in the production of soluble PHB synthase that led to the synthesis of PHB granules with no pigment. In another study by Akiyama et al. (2011), *Synechococcus* PCC7002 was transformed with PHA genes from *Cupriavidus necator* enhancing PHB production by up to 52% under heterotrophic conditions. In another study by Wang et al. (2013), synthetic metabolic pathways were constructed, and *Synechocystis* 6803 was standardized to synthesize (S)- and (R)-3-hydroxybutyrate (3HB) using CO_2_ directly. It was observed that *Synechocystis* cells easily secreted both forms of 3HB molecules without overexpressing the transporters. Furthermore, the competing pathway was suppressed by deleting the genes coding for PHB polymerase (*slr1829* and *slr1830*) from the genome of *Synechocystis*, which led to enhanced production of 3HB. The photosynthetic cultivation of recombinant *Synechocystis* TABd (for 21 days) produced 533.4 mg/L of 3HB. The accumulation of PHB is reported to increase under the conditions of nitrogen deficiency, and *sigE (*the Sigma factor) is also found to induce PHB synthesis and carbohydrate metabolic pathways. The *sigE* overexpression in *Synechocystis* 6803 improved PHB biosynthesis under nitrogen-deficient conditions. Interestingly, the monomer units and molecular weight of produced PHB are identical to those of PHB from the wild type (Osanai et al., 2013). Similarly, in another report, the recombinant *Synechocystis* sp. PCC 6803 obtained by transformation with the PHA synthase gene from *Cupriavidus necator* displayed enhanced activity of the PHA synthase enzyme; however, the total PHB content was not found to increase (Sudesh et al., 2002; Katayama et al., 2018). *Synechocystis* 6803 transformed with *pha* genes was found to be effective, and the recombinant cells had higher PHB content (12-fold high) in comparison to the wild type in response to nitrogen stress (Hondo et al., 2015). In *Synechocystis* 6803, phaAB overexpression and use of acetate (4 mM) were found to increase PHB content by 35% (DCW) (Khetkorn et al., 2016). In *Synechocystis*, overexpressing the acetoacetyl-CoA reductase gene improved the production of R-3-hydroxybutyrate to 1.84 g L^−1^, and maximum volumetrical production was 263 mgL^−1^ day^−1^ in *Synechocystis* (Wang et al., 2018). In another study, deletion of the *agp* gene encoding for ADP-glucose pyrophosphorylase from *Synechocystis* sp. PCC 6803 altered the partitioning of cellular carbon, and that consequently led to increased PHB production (18.6%), titer (232 mg/L), and productivity (7.3 mg/L/day) (Wu et al., 2002). Enzymes like acetyl-CoA hydrolase, phosphotransacetylase, and phosphoketolase control the level of acetyl-CoA in microbial cells. Acetyl-CoA can be converted into a large number of compounds based on the cell's requirement by employing different enzymes, particularly those that are used for PHB biosynthesis. Phosphotransacetylase (*pta* gene) and acetyl-CoA hydrolase (*ach* gene) are enzymes that convert acetyl-CoA into acetate. Phosphoketolase (encoded by *xfpk* gene) is an enzyme that, on the other hand, increases the level of acetyl-CoA in cells. Carpine et al. (2017) used a different strategy to amplify the production of PHB. For this experiment, recombinant *Synechocystis* PCC 6803 was designed by engineering central carbon metabolism instead of overexpressing or introducing PHB synthesis genes such that the PHB synthesis pathway gets downregulated and acetyl-CoA level increases. For this, seven diverse mutants were constructed that harbored individually or in the arrangement of three dissimilar genetic modifications to the metabolic pathway of *Synechocystis*. These included deletions of acetyl-CoA hydrolase-*Ach* and phosphotransacetylase-*Pta* and expression of heterologous phosphoketolase-*XfpK* (*Bifidobacterium breve)*. It was observed that a strain having all the three recombinations in consolidation, i.e., *xfpk* overexpression in a background of double deletion (*pta, ach*), displayed a maximum PHB production of 12% PHB yield, titer of 232 mg/L, and productivity of 7.3 mg/L/day. Koch et al. (2020) engineered a strain that lacked PirC (the regulatory protein, product of sll0944) that exhibited high phosphoglycerate mutase activity and increased level of PHB in response to nutrient stress. This was further modified to produce more PHBs by transferring *phaA* and *phaB* (PHA metabolism genes) from a PHB-synthesizing bacterium, *Cupriavidus necator*. This strain was termed as PPT1 (ΔpirC-REphaAB), and the production of PHB was found to be high (constant light as well under day-night conditions). Under nitrogen- and phosphorus-deficient conditions, it produced 63% of PHBs, and this level further increased by 81 percent when acetate was added (under identical culture conditions). The PHB production achieved by PPT1 was the maximum ever documented for any known cyanobacteria. Thus, the above studies indicate that genetic modification can be a promising tool in cyanobacteria to achieve sustainable and cost-effective PHB production on an industrial scale. Some of the wild-type and recombinant cyanobacterial strains used for biosynthesis of the polymer are listed in Table 2.

**Table 2:** List of wild-type and recombinant cyanobacterial strains used for the production of polymers.

**Cyanobacterial strains**	**PHB content (% DCW)**	**Substrate and culture conditions**	**PHA composition**	**References**
*Spirulina platensis*	6.0	CO_2_	PHB	Campbell and Balkwill, 1982
*Spirulina maxima*	7–9	CO_2_, N and P limitation	PHB	De Philippis et al., 1992
*Synechocystis* PCC 7942	3.0	CO_2_, N limitation	PHB	Takahashi et al., 1998
	25.6	Acetate, N limitation		
*Synechocystis* sp. PCC 6803	38	Phosphate deficiency, gas exchange limitation, acetate, fructose	PHB	Panda and Mallick, 2007
	9.5	Photoautotrophic, N-limitation		
	11.2	Photoautotrophic, P-limitation		
*Arthrospira platensis* UMACC 161	1.0	CO_2_	PHB	Toh et al., 2008
	10	Acetate, CO_2_, N starvation		
*Nostoc moscorum* Agardh	60	Acetate and Valerate, N deficiency	PHB-co-PHV	Bhati and Mallick, 2012
*Nostoc moscorum*	22	CO_2_, P starvation	PHB	Haase et al., 2012
*Aulosira fertilissima* CCC444	85	Citrate and Acetate, P deficiency	PHB	Samantaray and Mallick, 2012
*Aulosira fertilissima* CCC 444	76.9	Fructose, Valerate	P (3HB-co-3HV)	Samantaray and Mallick, 2014
	65.7	Fructose, Valerate, P deficiency		
*Caltorix scytonemicola* TISTR 8095	25	CO_2_, N deficiency	PHB	Kaewbai-Ngam et al., 2016; Monshupanee et al., 2016
*Synechocystis* sp. PCC 6714	16	CO_2_, N and P limitation	PHB	Kamravamanesh et al., 2017
*Synechococcus elongates*	17.2	CO_2_, sucrose, N deficiency	Not specified	Mendhulkar and Laukik, 2017
*Synechocystis* sp. CCALA192	12.5	CO_2_, N limitation	PHB	Troschl et al., 2018
**Recombinant cyanobacterial strains used for the production of the polymer**				
*Synechococcus* sp. PCC 7942	25.6	Acetate, nitrogen limitation	PHB	Takahashi et al., 1998
*Synechocystis* sp. PCC 6803	11	Acetate, nitrogen limitation	PHB	Sudesh et al., 2002
*Synechococcus* sp. PCC 7942	1.0	CO_2_	PHB	Suzuki et al., 2010
*Synechocystis* sp. PCC 6803	14	Direct photosynthesis	PHB	Lau et al., 2014
*Synechocystis* sp. PCC 6803	7.0	CO_2_	PHB	Hondo et al., 2015
*Synechococcus* sp. PCC 7002	~4.5%	Light, CO_2_ (photoautotrophy)	P (3HB-co-4HV)	Zhang et al., 2015
*Synechocystis* sp. PCC 6803	26	CO_2_, N deprivation, photoautotrophic	PHB	Khetkorn et al., 2016
	35	Acetate, N deficiency		
*Synechocystis* sp. PCC 6803	12	CO_2_, photoautotrophic	PHB	Carpine et al., 2017
*Synechocystis* sp.	35	CO_2_	PHB	Wang et al., 2018

## Challenges and Constraints in Production of Biofuel and Bioplastics From Cyanobacteria

Challenges at different steps in large-scale production appear, because outdoor cultivation may vary from ideal indoor conditions. These might occur at basic steps like insufficient light or nutrients, contamination in open pond cultivation systems, or the extraction, purification, and harvest stages. At times, the harvest is not efficient enough to be scaled up at a commercial level. Therefore, there is always a need for competent cyanobacterial strains by manipulation of metabolism to enhance fuel and bioplastic production and minimize raw material inputs and cost. There are multiple bottlenecks at various steps including operations and logistics, which need to be addressed to get the desired quality and quantity of biofuel and bioplastics.

### High Inputs of Resources (Space, Nutrients and Water)

The impact will directly be seen on land use pattern and agricultural inputs if the acceptance of algal biofuels increases in the near future. Preparedness for a future where acceptance of eco-friendly fuels will become imperative needs a well-planned cultivation model (Nozzi et al., 2013). Investment for setting up the initial infrastructure for the development of cost-effective scaling-up and harvest technology needs an upgrade.

### Technical and Economic Limitations

These include maintenance of optimal growth conditions throughout the culture until the harvest, fine-tuning the metabolic needs of strains, various aspects of boosting biosynthesis efficiency, biomass production (Lau et al., 2015), and efficient pre-treatment optimization with least emissions and high yield (Khan et al., 2018) along with the process of dewatering (Anyanwu et al., 2018). Other issues include analysis of nutrient consumption rate and its flux and continuous mining of efficient indigenous strains with better photosynthetic efficiency and higher productivity.

As discussed in many research studies, the use of native and genetically engineered heterotrophic microorganisms (*Bacillus megaterium, Cupriavidus necator, Escherichia coli, Pseudomonas aeruginosa, Ralstonia eutropha*, and *Saccharomyces cerevisiae*) has been the prime focus for producing PHAs (Pandian et al., 2010; Agnew et al., 2012; Ienczak et al., 2013). However, the fermentation process normally involves high production costs and hence limits their applicability for PHA production. This elevated cost is primarily due to the carbon source that is used to maintain the fermentation, productivity, downstream processing, and purification processes. It has been found that agricultural and industrial wastes along with other cheap carbon sources can help in lowering the cost of production, but this compromises the yield and requires redesigning of unit operation (Ienczak et al., 2013). There are studies indicating that the application of cyanobacteria in the production of industrial PHAs can help in lowering the cost of nutritional inputs as they have fewer nutritional requirements in contrast to heterotrophic bacteria (Asada et al., 1999; Sharma et al., 2007). Thus, PHA production *via* cyanobacteria would be effective in producing biodegradable bioplastics by sequestering CO_2_ as a costless source of carbon and thus can significantly contribute in lowering PHA production costs. However, these cyanobacteria normally give lower yield than heterotrophic organisms. Therefore, extensive inputs by research and development are required to upscale the yield of using cyanobacteria for industrial purposes.

### Transitioning From Pilot Phase to Industrial-Scale Production

In the urge of rising global energy and economic crises, cyanobacteria are emerging as a potential source for biofuels and bioplastics. Various studies are going on to take these bio- factories from laboratories to industries, and extensive research is focused on industrial upscale of a large variety of products, especially biofuels and bioplastics, from cyanobacteria under outdoor conditions. Additionally, many products like pigments can also be derived from the leftover residue after PHB extraction and can be used in a wide variety of downstream processes (Forchhammer and Koch, 2022). Along with industrial-level advantages, using cyanobacteria can help in reducing the cost related to production, increasing yield, managing waste, and producing energy-rich livestock that can, therefore, lead to sustainable production and environmental conservation (Gomes Gradíssimo et al., 2020). Many research studies have strongly recommended the development and use of improved recombinant strains of cyanobacteria that can accumulate high levels of acetate and polymer either by gene silencing or gene transfer. Additionally, whole production or reactor units are required to be innovatively redesigned. By this, several challenges related to low production of polymer can be solved (Drosg et al., 2015). However, with increasing and uncertain demands and supplies, industries are yet to report to derive maximum profits as compared to their high operational costs, and greenhouse gas emission and contamination during production of bioplastics on a large-scale industrial level pose challenges in using cyanobacteria for production of biopolymers (Priyadarshini et al., 2022; Rosenboom et al., 2022; Samadhiya et al., 2022). Interestingly, more focus on simultaneous degradation of petrochemical plastics and production of biodegradable plastics could be a promising approach (Priyadarshini et al., 2022).

### Marketing and Acceptance

Once the product obtained manages the scope and implementation inconsistencies, various hurdles need to be surpassed in order to reach economically feasible quality and quantity of biofuel (Gupta et al., 2020). These hurdles include the cost barriers, trade limitations, and issues with engines' fuel interface to meet the standards of global energy demand (Rodionova et al., 2017).

## Advancements, Future Perspectives, and Novel Approaches for Eco-Friendly Cyanobacterial Biofuel and Bioplastic Production

To overcome the abovementioned challenges and scale up biofuel production and use, strategies for overcoming each hurdle need to be optimized. In comparison to terrestrial plants, their tiny genome size, short development cycle, and simple metabolic activities have made their genetic manipulation more efficient (Singh et al., 2017). A huge amount of data on cyanobacterial genome sequencing has aided in the building of genome-scale models (GSMs) for a wide range of species, from model species *viz. Synechocystis* sp. PCC6803 to industrially promising strains like *Arthrospira platensis* NIES-39 (Yoshikawa et al., 2015). As a result, cyanobacterial genetics and metabolic control systems are elaborately characterized. This information has opened new vistas for genetic engineering of cyanobacteria to make biofuels. Cyanobacteria are employed as microbial model systems to focus on production of various biofuel forms. Strategies and improvements on genome-scale networks and modeling are enhanced and used for efficient cyanobacterial-based biofuels using the systems metabolic engineering method (Klanchui et al., 2016).

Ever-progressing genomics and systems biology have resulted in the recognition of new paradigms for systems metabolic engineering. Because of breakthroughs in metabolic engineering, the industrial production of cyanobacterial biofuels has recently become a reality. To capitalize on the usage of these organisms for better biofuel production, strategies to improve lipid productivity, salt tolerance, and other value-added parameters or design features have been devised (Sitther et al., 2020). Cultivation efficiency is observed to be maximized by the addition of waste paper (which enhances degradation) and co-cultivation/co-culture of autotrophic cyanobacteria with heterotrophic bacteria (which adds secondary metabolites to the cultivation system) (Luan and Lu, 2018).

Cyanobacteria have a great potential in the field of industrial biotechnology, which deals with the manufacture of a broad variety of bio-products such as hydrogen, isoprenoids, alcohols, and other high-value bioactive substances. Several innovative strategies have recently been applied in the discovery and development phases for the commercial application of cyanobacteria.

The demand for polymers in developed countries is expected to rise by 2–3 folds, and a lot of current studies are largely focused on finding a substitute to synthetic plastics. Heterotrophic bacteria produce 17–90% (DCW) of PHBs depending on nutrient supply and growth conditions. However, heterotrophs require carbon from heavily irrigated arable lands, and a huge cost is involved in the fermentation of heterotrophic bacteria, thus limiting their application in large-scale production of PHBs (Balaji et al., 2013). Cyanobacteria possess the ability to synthesize PHAs under mixotrophic and photoautotrophic growth conditions when supplemented with substrates such as acetate, propionate, and glucose. They can serve as an alternative for the biosynthesis of PHB, as 50% of the biomass of cyanobacteria is made of carbon and they utilize carbon sources from industrial effluents that permit the assimilation process by photo-biorefineries. The cyanobacterial cultivation in open systems like the one conducted for *Spirulina* sp. may further help in reducing the cost, as these systems are easy to build and function (Klinthong et al., 2015; Slocombe and Benemann, 2017; Meixner et al., 2018). The isolation and purification of PHBs from cyanobacteria are almost same as those conducted for PHA production *via* Gram-negative heterotrophic bacteria. Thus, by simplifying the design of photobioreactors, the applied material and decreasing the energy consumption can help in reducing the cost of bioreactors and subsequently will further lessen the cost of cyanobacterial PHB production (Drosg et al., 2015; Singh and Mallick, 2017; Costa et al., 2018). Besides this, other factors that contribute to increased cost of production are the cost involved in the procurement of raw material and extraction and purification processes. The method for PHAs recovery from the cell biomass plays a considerable role in the quality of PHA materials obtained as well as the cost involved in the process. Thus, various innovative methods like quick extraction methods for biomass disruption *via* enzymatic or chemical, mechanical methods, and high temperature and pressure for cell disruption are required for studying various PHA-synthesizing archaeal and eubacterial species (Madkour et al., 2013; Riedel et al., 2013; Samorì et al., 2015). Moreover, the costs involved in the procurement of raw materials for cyanobacterial cultivation surpass 50% of the entire expenditure involved in the production of PHBs *via* the heterotrophic method of production. The carbon source corresponds to 60% of the total expenditure on nutrients required for the cultivation of cyanobacteria under autotrophic conditions. Thus, to decrease the expenditure, there is a need to find a cheap alternative of carbon or nutritional sources like agro-industrial waste, carbon enriched wastewater, CO_2_ from cement plants, oil refineries, and thermoelectric combustion gas for cyanobacteria or heterotrophic bacteria. In addition to this, the use of residues and cheap nitrogen sources for culture like ammonium salts, urea, and nitrogen-enriched wastewater can further decrease the cost of nutrients (Borges et al., 2013; Costa et al., 2018).

Thus, there is a need to modify the cultivation, harvesting, and extraction methods, develop improved photobioreactor, find environment friendly and cost-effective recovery substitutes or downstream processes for cyanobacterial PHAs that can significantly boost PHA productivity and thereby would facilitate lowering the price associated with it (Singh and Mallick, 2017; Costa et al., 2018). In addition to this, the application of metabolic inhibitors has also been recommended to improve cyanobacterial PHA yield. The optimization of PHB production in cyanobacteria can be achieved by isolating strains that exhibit high capability of PHB biosynthesis. Genetic engineering is reported to be one of the promising methods to achieve cost-effective and sustainable PHA production from cyanobacteria. Cyanobacterial strains can be engineered with PHB synthase, acetoacetyl-CoA reductase, 3-ketothiolase genes, etc (Chen and Wu, 2005; Tao et al., 2008). Furthermore, the identification of SigE (RNA polymerase sigma factor) (Rre37), response regulator and presence of cyanobacterial entire Krebs cycle is found to be of great value in increasing the PHA synthesis in cyanobacteria (Singh and Mallick, 2017). Thus, cyanobacteria offer a promising photosynthetic platform to enhance PHA production. This has received immense attention as a result of current advancements in cultivation and metabolic engineering methods (Oliver et al., 2016).

Exploring the cyanobacterial “omics” research and technology is a current achievement that includes advancements in genetic modification and synthetic biology. These methodologies aided in the creation of complex metabolic engineering programs aimed at creating strains tailored for specific industrial biotechnology applications. Furthermore, emerging approaches such as Clustered Regularly Interspaced Short Palindromic Repeats associated protein (CRISPR/Cas) and CRISPRi (CRISPR interference) study in cyanobacteria (Gale et al., 2019) hold a lot of promise. To evaluate and design cyanobacteria for increased production, a well-studied model system and integration of synthetic biology approaches like metabolic models and genomic models, omics approach and genetic tools, and use of genetic manipulation are required to achieve enhanced bioplastic production (Santos-Merino et al., 2019). These models have emerged as a result of high-throughput omics investigation in cyanobacteria, which includes the use of practically applicable omics technologies in *Synechocystis*, a representative strain for cyanobacteria. Strategy on a large scale requires the expression of several genes that, in turn, has led to the innovation of more competent genetic tools in recent years like a system based on CRISPR (Santos-Merino et al., 2019). With the completion of a CRISPRi-gene repression library and a novel enhanced natural transformation method in cyanobacteria, the prospective execution of research advancements for production by biosynthesis can be hastened (Pope et al., 2020). The results of more extensive techniques and technology are encouraging in terms of establishing efficient cyanobacterial production systems. CRISPR/Cas tools are known to produce a large number of mutant knock-in and knock-out strains with desired traits (Sreenikethanam and Bajhaiya, 2021). These gene-editing tools are yet to be extensively investigated in algal systems, predominantly in the field of bioplastic research. It is also suggested that increasing the basic understanding related to cellular structure, metabolism, photosynthesis of cyanobacteria, and application of molecular tools can help in increasing PHA biosynthesis and productivity, as the *Synechocystis* sp. PCC 6803 genome sequence is available and there are various molecular and systems biology methods accessible to study this bacterium. Biosynthetic pathway engineering requires an in-depth understanding of cellular metabolism. High-throughput omics tools have been applied to understand the dynamic process of *Synechocystis* 6803 in response to several physiological conditions (Yu et al., 2013). Approaches based on synthetic biology to identify as well as develop new, fast-growing, tolerant cyanobacterial strains by simultaneously and critically considering genetic transformation, computational biology aspects, and scale-up techniques are continually being pursued to fast-forward the mass production of efficient cyanobacterial biofuels (Liang et al., 2018).

## Conclusions

Known as blue-green algae, prokaryotic, photosynthetic, Gram-negative organisms, cyanobacteria are of immense biological value. Among the various alternatives to reduce GHG emission by the usage of low carbon-based fuels, biofuels and bioplastics can help in lessening the atmospheric carbon footprint and can further lessen the oil dependence on finite resources for sustainable growth and development. The circular economy suggests the pattern of production and consumption by reducing the demand, reusing, repairing, recycling, and resource extraction or recovery from existing materials and their products for a long time. By this, the use of cyanobacteria in deriving biofuels and bioplastics using metabolites in different industries can be of help in the circular economy and achieve sustainable goals as suggested by Rosenboom et al. (2022). Most of the applications of cyanobacteria for sustainable production are still marked by low product yield. In this regard, computational methods for scheming and designing a suitable strain based on genome-scale metabolic models hold an enormous potential to appreciably enhance product yield. Integration of progressing system biology and synthetic biology would help in finding and developing new and economical methods to achieve eco-friendly, cost-effective, and sustainable biofuel and bioplastic production from cyanobacteria.

## Author Contributions

PA, RS, PK, AM, and RM helped in drafting, writing, and editing manuscript, including figures and table. JP and SS helped in making figures and tables and were involved in editing. GS generated the idea, designed the manuscript, and involved in writing and editing manuscript. All authors contributed to the article and approved the submitted version.

## Conflict of Interest

The authors declare that the research was conducted in the absence of any commercial or financial relationships that could be construed as a potential conflict of interest.

## Publisher's Note

All claims expressed in this article are solely those of the authors and do not necessarily represent those of their affiliated organizations, or those of the publisher, the editors and the reviewers. Any product that may be evaluated in this article, or claim that may be made by its manufacturer, is not guaranteed or endorsed by the publisher.
